# Introduction to the annual issue on “Infant Hydrocephalus“

**DOI:** 10.1007/s00381-021-05305-6

**Published:** 2021-08-19

**Authors:** Abhaya V. Kulkarni, Ulrich-Wilhelm Thomale

**Affiliations:** 1 Division of Neurosurgery, Hospital for Sick Children, University of Toronto, Toronto, Canada; 2 Pediatric Neurosurgery, Campus Virchow Klinikum, Charité Universitaetsmedizin Berlin, Augustenburger Platz 1, 13353 Berlin, Germany

Hydrocephalus itself is the downstream effect resulting from a plethora of underlying conditions that disturb CSF circulation. The age at which the condition presents affects the clinical presentation and impacts the treatment options and decision making. About three quarters of patients will present during infancy, i.e., before 24 months of age. Treatment decisions made during infancy will have reverberating lifetime impact on the child and the future adult, so there is a heavy weight on the neurosurgeon and the family to make decisions that are well thought out. Therefore, this Special annual issue, dedicated entirely to infant hydrocephalus, is of great importance to the pediatric neurosurgery community.

The development of CSF shunts in the 1950s dramatically and indelibly improved the treatment of infant hydrocephalus and is one the most important contributions to neurosurgery of the last 100 years. While endoscopy has provided a viable alternative treatment in some cases, CSF shunt, along with its many potential complications, remains the most common form of treatment.

In the last several years, however, we have seen significant progress in our understanding of hydrocephalus pathophysiology and the treatment options available. This Special annual issue provides a current perspective on hydrocephalus epidemiology, general pathophysiology, genetic alterations, inflammatory mechanisms, and the role of ciliary function in CSF dynamics. Additionally, the nuances involved in treating hydrocephalus associated with specific conditions, such as tumors, hemorrhage, infection, spina bifida, encephalocele, cysts, venous congestion, and craniosynostosis, are addressed. Aside from further consideration of shunt complications and valve selection, the Special issue also includes discussion of newer surgical techniques such as choroid plexus cauterization, endoscopic third ventriculocisternostomy, and neuroendoscopic lavage.

These papers have been contributed by leaders in hydrocephalus research and treatment from around the world. We are very grateful to each of them for contributing their expertise to this Special issue.

There is, of course, much work that remains to be done to improve infant hydrocephalus treatment. Future work will need to focus on individualizing treatment modalities for hydrocephalus to enable the best possible neurodevelopment outcome for every child, which remains the most important treatment target of all.

